# What People Believe about How Memory Works: A Representative Survey of the U.S. Population

**DOI:** 10.1371/journal.pone.0022757

**Published:** 2011-08-03

**Authors:** Daniel J. Simons, Christopher F. Chabris

**Affiliations:** 1 Department of Psychology, Beckman Institute, University of Illinois, Champaign, Illinois, United States of America; 2 Department of Psychology, Union College, Schenectady, New York, United States of America; Yale University, United States of America

## Abstract

Incorrect beliefs about the properties of memory have broad implications: The media conflate normal forgetting and inadvertent memory distortion with intentional deceit, juries issue verdicts based on flawed intuitions about the accuracy and confidence of testimony, and students misunderstand the role of memory in learning. We conducted a large representative telephone survey of the U.S. population to assess common beliefs about the properties of memory. Substantial numbers of respondents agreed with propositions that conflict with expert consensus: Amnesia results in the inability to remember one's own identity (83% of respondents agreed), unexpected objects generally grab attention (78%), memory works like a video camera (63%), memory can be enhanced through hypnosis (55%), memory is permanent (48%), and the testimony of a single confident eyewitness should be enough to convict a criminal defendant (37%). This discrepancy between popular belief and scientific consensus has implications from the classroom to the courtroom.

## Introduction

Do people think that memory works like a video camera? Do they believe that memories are immutable once they are formed? Answers to questions like these have important ramifications for psychologists and for the legal system. The “beyond the ken” standard [1] for admitting expert testimony in courts requires that experts provide information that goes beyond what jurors already know. If people generally understand how memory works, and common sense is consistent with expert consensus, then courtroom testimony by psychologists about memory may be unnecessary [1]. However, if popular beliefs contradict what science knows to be true, then memory experts will have an important role in educating juries, judges, lawyers, and the public about the properties of memory.

People often dismiss behavioral science research as merely recapitulating common sense, but many important psychological findings are counterintuitive. Some of the most striking discrepancies between popular intuitions and established science come from the study of memory. When people recall an emotionally charged event, they believe that their vivid memories are precise and accurate, largely because they rarely encounter evidence for distortions in their own memories. The surprise people express when they first realize how easily they can miss changes in their environment [2] or when they fail to notice when a person in a gorilla suit unexpectedly appears in a video [3] reflects incorrect underlying beliefs about the completeness of visual encoding, the richness of visual representations, and the exhaustiveness of visual awareness [4], [5].

Although several studies have documented the pervasiveness of mistaken beliefs about memory in the laboratory and classroom [5], [6], only a few studies have examined the prevalence of mistaken intuitions about memory in the broader population. In one study, students and jury pool members underestimated the influence of factors known to affect eyewitness accuracy, including the status of the person providing post-event suggestions, the age of the witness, the delay between the original event and recall, and the impact of providing warnings about potentially misleading suggestions [7]. In another study, jury pool participants were also found to hold mistaken beliefs about memory, lending support to the idea that members of the public do not concur with the expert consensus view of memory [8]. In contrast, a series of surveys using convenience samples of the general public in Vancouver, Canada shopping malls suggested relatively good agreement with expert consensus about eyewitness accuracy [9].

To our knowledge, only one large-scale study of the general public has examined beliefs about the properties of memory [1]. That study examined how well prospective jurors in the Washington, DC jury pool understood the results of memory research about eyewitness testimony. Although this jury-pool survey focused mostly on beliefs about factors influencing eyewitness memory, it included some true/false items about the nature, precision, and permanence of memory. For example, one item stated, “The act of remembering a traumatic event is like a video recording in that one can recall details as if they had been imprinted or burned into one's brain.” The survey recorded high rates of incorrect responses to these items, suggesting that many potential jurors misunderstand the properties of memory.

Most of these surveys focused on eyewitness accuracy, not the properties of memory more broadly. And all previous studies were limited in sample size, geography, and/or demographic representativeness. Here we report the results of the first large-scale, nationally representative survey designed to measure intuitive beliefs about the properties of memory. Our study is not a general survey of misconceptions about all aspects of human behavior. Nor is it an exhaustive survey of beliefs about memory. Although we also asked about a variety of beliefs about the mind, here we report on a subset of the questions that focused on beliefs about memory. These items were selected because we expected that they might reveal a divergence between expert consensus and commonly held beliefs and because they hold both theoretical and practical significance.

## Methods

### Subjects

We hired SurveyUSA to poll a nationally representative sample of the United States population during a one-week period in June 2009 (the “public sample”). Each of the 1838 respondents was assigned a weight based on the Census-derived U.S. population demographics for sex, region, age, and race to produce a nominal, demographically-representative sample size of 1500 people (Table 1). Respondents also reported their education, income level, number of psychology courses ever taken, and number of psychology books read in the past three years. This research was conducted with the approval of the IRB of the University of Illinois (protocol #09714). All data were collected anonymously, and the study conformed to the guidelines and principles of the Declaration of Helsinki. The protocol was given a waiver of consent given that it is an anonymous survey.

**Table 1:** Analyses of memory knowledge as a function of demographic measures for public sample respondents.

Category [test statistic]	Subcategory	% of Sample	Average # correct (out of 6)
**Age** [*t*(1496) = 3.71, *p*<.001]	<50 years	56.7	2.50
	≥50 years	43.3	2.21
**Gender** [*t*(1496) = 0.75, *p* = .453.]	Male	48.3	2.40
	Female	51.7	2.34
**Race** [*F*(4,1493) = 3.88, *p* = .004]	Hispanic	11.0	2.22
	Black	11.1	2.02
	White	72.0	2.43
	Asian	1.4	2.59
	Other	4.5	2.58
**Education** [*F*(3,1483) = 27.22, *p*<.001]	Grad School	18.6	2.90
	College Grad	17.5	2.50
	Some College	40.9	2.34
	No College	22.9	1.88
**Income** [*F*(2,1429) = 25.80, *p*<.001]	<$40K	36.3	2.06
	$40K–$80K	36.7	2.44
	>$80K	25.3	2.74
**Region** [*F*(3,1494) = 2.28, *p* = .077]	Northeast	19.4	2.38
	Midwest	22.7	2.36
	South	35.7	2.27
	West	22.3	2.54
**Psychology Books** [*F*(3,149) = 5.38, *p* = .001]	0	40.3	2.30
	1	21.4	2.18
	2	17.3	2.59
	3+	21.1	2.53
**Psychology Classes** [*F*(3,149) = 14.33, *p*<.001]	0	37.3	2.21
	1	18.6	2.08
	2	16.2	2.52
	3+	27.9	2.70

The percentages for sex, race, and age (older/younger than 50) resulting from the weighting process are matched to U.S. Census values. After weighting the nominal sample size is 1500 respondents. The percentages for education, income, number of psychology books read, and number of psychology classes taken were measured but not used to determine sampling weights. Note that some subjects declined to answer the income question (4.4%) and the education question (0.07%).

### Survey items

The survey began with the following script: “This is SurveyUSA, conducting a short opinion survey that will help scientists better understand how the brain works. We don't need your name, and everything you tell us is confidential. To start, press 1. [if no response, continue after 5 seconds] This is the opinion research company SurveyUSA calling to include you in scientific research about how the mind and brain work. There is no cost, we do not need your name, and everything you tell us is confidential. You'll be on the phone for just a few minutes. To start, press 1 on your touchtone phone, now.” After subjects pressed 1, the script continued, “Great, let's get started.” For each item, subjects were asked to respond “strongly agree,” “mostly agree,” “mostly disagree,” “strongly disagree,” or “don't know” using the keys on their phone.

The survey consisted of 16 substantive statements followed by demographic questions. The survey items were based on similar items in a number of sources (e.g., [1], [5], [6], [10], [11]) and were chosen because they would measure what we anticipated would be widespread misconceptions. Each statement was worded to be inconsistent with the scientific consensus, so that the percentage of respondents agreeing would reflect the percentage of the public holding a mistaken belief.

Items were worded to be interpreted consistently by experts and laypeople, and were reviewed by SurveyUSA to ensure comprehensibility. The script was written at a 7th-grade reading level. We deliberately chose items for which we expected a discrepancy between scientific consensus and popular belief, so our results should not be taken to indicate the full scope of psychological understanding by the general public.

Here we report results for six items related to beliefs about the properties of memory (see Table 2). Other survey items addressed beliefs about other topics in psychology (e.g., the myth that people use only 10% of their brain, the belief that you can feel someone staring at the back of your head, the idea that listening to Mozart increases IQ, etc.), and the data from these are not reported here. The memory items were interspersed among the other items.

**Table 2:** Statements used and percentage of respondents giving each response, with the expert (*N* = 16) and the full Psychonomics sample (*N* = 73) percentages given for comparison.

Statement	Group	Strongly Agree	Mostly Agree	Mostly Disagree	Strongly Disagree	Don't Know
**Amnesia**: *People suffering from amnesia typically cannot recall their own name or identity.*	**Public**	47.8	34.9	10.1	3.7	3.7
	**Experts**	0.0	0.0	12.5	87.5	0.0
	**Psychonomics**	0.0	1.4	31.5	57.5	9.6
**Confident testimony**: *In my opinion, the testimony of one confident eyewitness should be enough evidence to convict a defendant of a crime.*	**Public**	11.2	25.9	35.1	24.7	3.1
	**Experts**	0.0	0.0	6.2	93.8	0.0
	**Psychonomics**	0.0	0.0	11.0	87.7	1.4
**Video memory:** *Human memory works like a video camera, accurately recording the events we see and hear so that we can review and inspect them later.*	**Public**	23.9	39.1	23.4	11.3	2.4
	**Experts**	0.0	0.0	6.2	93.8	0.0
	**Psychonomics**	0.0	0.0	2.7	97.3	0.0
**Hypnosis:** *Hypnosis is useful in helping witnesses accurately recall details of crimes.*	**Public**	15.0	39.6	26.9	10.4	8.1
	**Experts**	0.0	0.0	18.8	68.8	12.5
	**Psychonomics**	0.0	0.0	15.1	69.9	15.1
**Unexpected events:** *People generally notice when something unexpected enters their field of view, even when they're paying attention to something else.*	**Public**	27.2	50.3	18.3	2.1	2.1
	**Experts**	0.0	18.8	31.2	50.0	0.0
	**Psychonomics**	2.7	15.1	35.6	43.8	2.7
**Permanent memory:** *Once you have experienced an event and formed a memory of it, that memory does not change.*	**Public**	16.5	31.1	34.7	14.1	3.6
	**Experts**	0.0	0.0	0.0	93.8	6.2
	**Psychonomics**	0.0	0.0	6.8	91.8	1.4

Each question has been given a short label (in bold) for ease of exposition in the text. Numbers corresponding to each item for the public sample represent the percentage of the weighted respondents giving each response.

### Procedure

Respondents were selected using random-digit dialing of landline phone numbers within area codes that represented all regions of the United States. Approximately 42.4% of the 79,014 attempted calls were answered by a human being who responded to at least one question. Approximately 5.5% of these calls resulted in a completed survey (thus, 2.3% of all dialed numbers yielded a completed survey). SurveyUSA uses “robotic polling,” a technique in which the recorded voice of a female announcer reads each statement and respondents press keys on their telephone to answer. Robotic polling provides a more consistent subject experience than live-operator polling because each participant hears the statements in the same voice, regardless of their own race, income, ethnicity, region, etc. People may also be less affected by social demands when responding to a recorded voice than a person. The relatively low response and completion rates are fairly typical for robotic polling, but low response rates are not indicative of inaccurate polls. Robotic polls with comparable response rates have been among the most accurate political polls in recent election cycles (see www.fivethirtyeight.com for detailed analyses of polling accuracy).

### Expert validation

We confirmed the expert consensus for our survey items by polling attendees at a session on meta-memory at the 2010 meeting of the Psychonomic Society (the “expert sample”). Before the session started, we distributed 200 single-page surveys which we then collected before our presentation. This survey included just the six memory items and gave participants the same five response options (except that “Don't Know” was relabeled “Don't Know/Unclear”). Respondents also reported their academic status (professor, postdoc, grad student, undergrad, other), indicated whether they conduct research on memory (yes or no), and—if yes—for how long they have done so (0–5, 5–10, or >10 years).

A total of 73 attendees (27 faculty, 9 postdocs, 26 graduate students, 3 undergraduates, and 1 research scientist) completed surveys prior to the presentation. To ensure that our validation was based on a truly expert consensus, we restricted our expert sample to professors with more than 10 years of memory research experience. (As shown in Table 2, though, the full sample of 73 respondents produced the same response pattern as the experts.)

## Results and Discussion

All summary statistics (e.g., percent agreement) and analyses were conducted by weighting each respondent in the public sample to account for over- or under-sampling of their demographic group. Only respondents who completed all the substantive questions as well as the demographic questions about their sex, race, and age were included in the analyses because those responses were needed to weight the respondent's answers appropriately. Non-responses to the remaining demographic questions were treated as missing data.

For simplicity of analysis and interpretation, we combined “strongly agree” and “mostly agree responses” into an “agree” category and did the same for “disagree” responses. Given that subjects could respond “don't know” to any substantive question, agree or disagree responses presumably represent respondents' true opinions. Public sample subjects averaged 0.23 “don't know” responses out of six possible responses (Median  = 0, SD  = 0.63); only 5.3% of subjects gave more than one “don't know” response, and 84.7% did not use this option at all. The average “don't know” response rate across items was 4.2% (SD  = 2.2%, range  = 2.1–8.1%). The overall means for each item reflect the percentage of “agree” responses in the entire sample of 1500 respondents, including those who said “don't know.” The analyses and means for subgroups (e.g., different levels of education) exclude “don't know” responses. Consequently, those percentages represent the number of “agree” responses among those subjects who gave either an “agree” or “disagree” response (see Table 3).

**Table 3:** Percentage of public sample agreeing with each statement, by level of education reported.

Statement	No college (*N* = 342)	Some College (*N* = 610)	College Graduate (*N* = 261)	Graduate School (*N* = 277)	Linear contrast
**Amnesia:** *People suffering from amnesia typically cannot recall their own name or identity.*	87.4	87.4	87.8	78.8	*t*(1431) = 2.8, *p* = .005
**Confident testimony:** *In my opinion, the testimony of one confident eyewitness should be enough evidence to convict a defendant of a crime.*	48.0	40.7	35.6	24.7	*t*(1439) = 6.7, *p*<.001
**Video memory:** *Human memory works like a video camera, accurately recording the events we see and hear so that we can review and inspect them later.*	78.2	69.4	55.6	46.7	*t*(1450) = 9.1, *p*<.001
**Permanent memory:** *Once you have experienced an event and formed a memory of it, that memory does not change.*	64.9	47.4	43.4	40.6	*t*(1431) = 6.0, *p*<.001
**Hypnosis:** *Hypnosis is useful in helping witnesses accurately recall details of crimes.*	68.4	59.9	56.6	50.2	*t*(1365) = 4.4, *p*<.001
**Unexpected events:** *People generally notice when something unexpected enters their field of view, even when they're paying attention to something else.*	83.5	76.1	78.0	82.9	*t*(1453) = 0.0, *p* = .996

The parentheses under each education category show the weighted number of respondents out of the 1490 who answered the education item (the equivalent of 10 weighted participants did not answer this item).

For all but one of the six statements, education was negatively correlated with agreement: Respondents with more education were less likely to “agree” (see Table 3). However, in all cases, substantial percentages of even the most educated respondents (graduate education) still agreed with the statement.

Across all six items, an average of 60.4% of respondents agreed with statements that the expert sample almost uniformly rejected. In other words, fewer than 40% of the responses agreed with the scientific consensus. More than half of respondents disagreed with the expert consensus on four or more items, and only 1.5% of subjects matched the experts on all six items (see Table 4). As for any survey item, it is possible to conceive of circumstances or special situations in which an item might be true (for discussion, see the caveats section at the end of the results). The uniformity of the expert responses to our survey suggests that experts agree on the most straightforward interpretation of each of our items item. And, the consistent differences between expert responses and those of our survey respondents highlights the discrepancy between expert consensus and common beliefs. Below we provide the results for each item separately, followed by a discussion of demographic trends in responding.

**Table 4:** Percentage of subjects giving each number of correct responses (out of six possible correct answers).

# of “disagree” responses	% of public sample	% of expert sample (*N* = 16)	% of full Psychonomics Sample (*N* = 73)	Binomial Probability
0 (all incorrect)	10.8	0.0	0.0	1.6
1	18.1	0.0	0.0	9.4
2	27.8	0.0	0.0	23.4
3	19.6	0.0	0.0	31.2
4	15.2	6.2	11.0	23.4
5	6.9	25.0	27.4	9.4
6 (all correct)	1.5	68.8	61.6	1.6

Binomial probability gives the odds of getting that many correct if chance is 50% on any given item.

### Amnesia: 82.7% of respondents agreed that “people suffering from amnesia typically cannot recall their own name or identity.” All 16 experts disagreed

The form of amnesia that most typically results from brain injury involves the loss of the ability to form and consolidate new long-term memories (e.g., [12]). Agreement with this statement perhaps best exemplifies the pervasive influence of popular media portrayals of amnesia. Although a few movies come close to an accurate portrayal of amnesia as the loss of the ability to form andconsolidate new memories (e.g., *Memento*), most depict amnesia as something more like a much rarer fugue state in which someone cannot remember who they are and suddenly take leave of their home and work (e.g., *The Bourne Identity,* or the documentary *Unknown White Male*). The persistence of this factual misunderstanding despite more than half a century of research on the mechanisms of memory loss reveals a need for better popular outreach to explain the deficits that can result from brain injury.

### Confident Testimony: 37.1% agreed that “in my opinion, the testimony of one confident eyewitness should be enough evidence to convict a defendant of a crime.” All 16 experts disagreed

The link between confidence and accuracy in eyewitness testimony is more complex than typically presented in psychology textbooks. You are more likely to be accurate when you are more confident in your memory than when you are less confident [13]. However, the link between confidence and accuracy *across* individuals is more tenuous [14], in part because people differ in their baseline levels of expressed confidence [15]. Consequently, most memory experts agree that an isolated expression of confidence is at best a limited predictor of memory accuracy [14], [16], [17]. When people are wrongly convicted of crimes and later exonerated by DNA testing, the primary evidence often came in the form of a confident, but faulty, eyewitness identification [18], [19]. Lawyers and judges need to be aware that a sizeable minority of a typical jury pool likely misunderstands the fallibility of eyewitness testimony and may rely too heavily on confident witness statements.

### Video Memory: 63.0% agreed that “human memory works like a video camera, accurately recording the events we see and hear so that we can review and inspect them later.” All 16 experts disagreed

This statement incorporates several erroneous beliefs. First, the idea that memory works like a video camera implies a level of completeness and accessibility of our representations that is inconsistent with known limits on visual perception and attention [2], [20]. Second, video recording implies a passive process in which the visual world is imprinted into memory. But decades of research have documented the influences of stored information on encoding and memory, arguing against the idea that memories are pure, bottom-up records [21], [22]; what is encoded depends on top-down goals and expectations. Finally, the idea that memory retrieval is akin to rewinding and replaying a tape contradicts the well-established idea that memory retrieval is a constructive process influenced by knowledge, beliefs, expectations, and schemas [22], [23].

This belief is perhaps the most relevant to the role of expert testimony on memory in legal cases. If jurors believe that memory works like a video camera, they will be more likely to trust witnesses who saw an event without realizing that different people may encode the same event differently or that memory can be distorted by subsequent events.

### Permanent Memory: 47.6% agreed that “once you have experienced an event and formed a memory of it, that memory does not change.” 15 experts disagreed and 1 responded “Don't Know/Unclear.”

Even those believing in a permanent memory trace (with failed recall resulting from interference) acknowledge that the trace could be strengthened or weakened based on later experiences [21]. Consistent with the idea that people interpret this statement as reflecting the permanence and immutability of memory, responses to this question were correlated with responses to the *video memory* item (*r* = .36); people who endorsed the idea of video memory were more likely to endorse the idea of permanent memory (see Table 5).

**Table 5:** Pearson correlations among survey items for the public sample.

	Amnesia	Confident Testimony	Video Memory	Permanent Memory	Hypnosis	Unexpected Events
Amnesia	1					
Confident Testimony	r = .046† (*N* = 1403)	1				
Video Memory	r = .125 (*N* = 1403)	r = .213 (*N* = 1426)	1			
Permanent Memory	r = .157 (*N* = 1402)	r = .109 (*N = *1407)	r = .364 (*N = *1419)	1		
Hypnosis	r = .128 (*N* = 1341)	r = .109 (*N* = 1345)	r = .180 (*N* = 1354)	r = .168 (*N* = 1356)	1	
Unexpected Events	r = .084 (*N* = 1419)	r = .060* (*N* = 1428)	r = .127 (*N* = 1442)	r = .155 (*N* = 1424)	r = .077** (*N* = 1357)	1

“Don't know” responses were treated as missing data. All correlations were significant at p<.001 unless otherwise noted:

†p<.10.

*p<.05.

**p<.01.

### Hypnosis: 55.4% agreed that “hypnosis is useful in helping witnesses accurately recall details of crimes.” 14 experts disagreed and 2 responded “Don't Know/Unclear.”

Although hypnosis can lead to more recall by encouraging people to adopt a more lenient criterion [24], it does not lead to more accurate recall (for overviews see [25], [26]). The popular support for hypnosis is somewhat surprising given the degree to which the courts already treat hypnosis-based recollections as untrustworthy. A larger proportion of the public sample responded “don't know” to this question than any of the other ones, perhaps reflecting a greater degree of uncertainty about how hypnosis works.

### Unexpected Events: 77.5% agreed that “people generally notice when something unexpected enters their field of view, even when they're paying attention to something else.” 13 experts disagreed and 3 agreed

Although a number of studies have revealed the failure to notice unexpected events when attention is focused on something else (e.g., [3], [27], [28]), there is little direct evidence for the frequency with which unexpected events are noticed in general, a fact that might explain why three experts agreed. Note that there still is a large disparity between the expert sample (81% disagree) and the public sample (77.5% agree) about what captures attention. Although on its surface, this item appears to focus on attention rather than memory, events can only be incorporated into explicit memory if they are noticed, and attention plays a central role in the encoding process. Agreement with the statement is consistent with evidence that undergraduates believe salient events will draw attention even when people are focused on other attention-demanding tasks [5], and it might partly explain why people often are surprised by demonstrations of inattentional blindness [3].

If juries and lawyers believe that a suspect “should have” noticed some event, they will tend to see claims of ignorance as deliberate attempts to deceive (see [29], [30], [31] for examples). Notably, unlike the other memory statements, agreement with this statement was not associated with increasing education (Table 3).

### Demographic analyses

To analyze which demographic measures are associated with mistaken beliefs, we computed a general memory knowledge index as [6 – (# of “agree” responses)]. Given that the items were not chosen to encompass the full range of memory processes, the memory knowledge index reflects only the prevalence of these particular mistaken beliefs. The items were not chosen to tap a single construct of memory knowledge, and they were not highly inter-correlated (see Table 5; Cronbach's alpha  = .49; mean inter-item *r* = .14).

Unsurprisingly given the large size of the public sample, general memory knowledge varied significantly among subgroups of almost all the demographic variables, but all subgroups agreed, on average, with more than half of the statements (Table 1). Increased education was associated with better knowledge, linear contrast: *t*(1483) = 8.9, *p*<.001. Respondents with post-college education outperformed those with college degrees or some college experience, who in turn outperformed those with no college (all pairwise comparisons are significant by Scheffé test at *p*<.05). Because education is likely correlated with other individual differences such as socioeconomic status and intelligence, this association does not provide causal evidence that education increases understanding of how memory works.

Specific background in psychology, as measured by the number of psychology books read, was associated with better knowledge as well, linear contrast: *t*(1494) = 3.3, *p* = .001. Those people who had read 2 or 3+ psychology books outperformed those who had just read 1 book, although those reading no books were not different from the other groups. Note that the survey did not constrain what counts as a psychology book, so this variable might not reflect knowledge of scientific psychology. The number of psychology classes taken also had a significant linear effect: *t*(1494) = 6.2, *p*<.001. Those who took 3+ psychology courses outperformed those who took just 2 classes, and both of those groups outperformed those who took 1 course or no courses. Again, these associations do not permit causal conclusions, but they are consistent with the idea that learning about psychology improves understanding of memory.

### Caveats

Our results should be considered in light of several caveats. First, we treat the current expert consensus as accurate, while acknowledging that expert opinion can change over time. For example, in the 1970s, emotionally charged events or “flashbulb” memories were thought to be accurately recorded and recalled [32], [33], whereas experts now understand that even vivid memories can be distorted. We assume that the current consensus reflects the substantial accumulation of evidence over recent decades rather than shifts in fashion.

Second, we assume that our public sample accurately reflects the beliefs of the American public even though our response and compliance rates were relatively low (albeit typical for robotic polling). Any survey with a response rate lower than 100% risks sampling an unusual subset of the target population—those who willingly respond to phone surveys may differ systematically from the remainder of the population. This critique applies to all polling methodologies because it is impossible to measure the beliefs of people who refuse to respond. SurveyUSA's record of reliable and valid prediction in its political polling suggests that their methods accurately sample public opinion despite relatively low response rates. And the demographics of our sample were weighted to match the United States census, so our survey is representative in its final sampling (more so than almost any other experimental method, especially those that use convenience samples of undergraduates; see [34] for discussion of the representativeness problem). More importantly, a number of survey firms have examined the predictive validity of their polling as a function of response rate by taking extraordinary measures to increase the response rate (e.g., by repeatedly calling or visiting the same home over a two-month period). The consistent result across those studies is that increasing the response rate does not increase the reliability or predictive validity of survey results [35].

Third, for almost any statement of fact, experts can imagine a context that would make the statement true rather than false. For example, a single confident witness might provide enough evidence for conviction if the witness happened to know the accused before seeing the crime or if the witness had extensive opportunities to observe the suspect. Constructing statements of fact that could not be interpreted differently under any set of assumptions would require so many hedges and qualifications that the item likely would not convey the intended meaning (or even be intelligible) to laypeople. We worded our items so that the most straightforward interpretation would be false, and the uniformity of our expert consensus suggests that the intended interpretation of our items was clear.

Finally, all of our items were worded as positive statements, and people might have felt some pressure to agree with some of the items, thereby inflating agreement rates. However, robotic polling decreases the usual social demand to agree with the (nonexistent) “person conducting the study.” Furthermore, the availability of four levels of agreement, as well as a “don't know” option, should minimize a default tendency to agree. And, with negative wording, some of our items would be nonsensical or would not represent facts about the mind. Finally, given the high rates of agreement we find for many of our items, bias alone is unlikely to explain large deviations from 0% agreement—the expected rate if people fully understand the properties of memory.

### Conclusion

Each of the beliefs documented in this survey runs counter to expert scientific consensus and reflects a fundamental misunderstanding of the way that memory works. In a sense, the results of this survey are disappointing: Many of the ideas we tested refer to scientific findings that have been established for decades. This discrepancy between science and popular beliefs confirms the danger of relying on intuition or common sense when evaluating claims about psychology and the mind [30], [36], [37]. Accordingly, scientists should more vigorously communicate established and uncontroversial results (alongside new and surprising findings) in a way that leads to broader public understanding. Teachers should also be cognizant that many of their students come to the classroom with basic misconceptions about psychology.

The prevalence of mistaken beliefs in the general public implies that similar misunderstandings likely are common among jurors [8] and could well lead to flawed analyses of testimony that involves memory. At least for these basic properties of memory, commonsense intuitions are more likely to be wrong than right. Expert testimony on these issues could well help to overcome such misinterpretations (although see [38]), and at a minimum, it could make jurors aware of some of the limitations of memory. Future research should examine how people acquire faulty intuitions about memory and why those intuitions persist in the face of contradictory scientific evidence.
